# Genetic characteristics of *Bacillus anthracis* isolated from northwestern China from 1990 to 2016

**DOI:** 10.1371/journal.pntd.0006908

**Published:** 2018-11-12

**Authors:** Huijuan Zhang, Enmin Zhang, Jinrong He, Wei Li, Jianchun Wei

**Affiliations:** 1 National Institute for Communicable Disease Control and Prevention, Chinese Center for Disease Control and Prevention, Beijing, China; 2 State Key Laboratory of Infectious Disease Prevention and Control, Beijing, China; 3 Collaborative Innovation Center for Diagnosis and Treatment of Infectious Disease, Hangzhou, China; Beijing Institute of Microbiology and Epidemiology, CHINA

## Abstract

Anthrax is a global re-emerging zoonotic disease and is an endemic disease in China, especially in rural regions. In this study, the general characteristics of human anthrax outbreaks that occurred in areas of northwestern China over the past decade have been described. Meanwhile, the genetic characteristics of *Bacillus anthracis* isolated from these areas from 1990 to 2016 were analyzed by means of canonical single-nucleotide polymorphism (canSNP) analysis and multilocus variable-number tandem repeat analysis (MLVA) with 15 markers. Five sublineages/subgroups, namely, A.Br.001/002, A.Br.Vollum, A.Br.Aust94, A.Br.Ames and A.Br.008/009, were detected by using 13 canSNP sites. All of the sublineages were found in Xinjiang province, while one sublineage was found in Shaanxi, two in Gansu, three in Qinghai and four in Inner Mongolia. However, the geographical distribution of the *B*. *anthracis* populations exhibited different canSNP characteristics from those of the strains isolated before 1990 in China. In contrast to previous data, the A.Br.Ames subgroup was also observed to be scattered from Inner Mongolia to other provinces. All 106 strains were assigned to 36 MLVA15 genotypes, and 21 of these types were first observed in this study. The strains collected from anthrax outbreaks in recent decade were classified as subgroups A.Br.001/002 and A.Br.Ames and identified as genotypes MLVA15-28, MLVA15-30, MLVA15-31, MLVA15-38, MLVA15-CHN3, and MLVA15-CHN18. By canSNP analysis and MLVA, we found that the diversification of MLVA genotypes and the geographical distribution of *B*. *anthracis* populations is gradually becoming balanced across northwestern China. This study also provides preliminary survey results regarding the population diversity of *B*. *anthracis* in China, which will help promote the prevention and control of this important disease.

## Author summary

In this study, the general characteristics of human anthrax outbreaks that occurred in northwestern China over the past decade were described. Meanwhile, the genetic characteristics of *Bacillus anthracis* isolated from these areas from 1990 to 2016 were analyzed with the canSNP and MLVA15 methods. Our results showed a diversity of MLVA genotypes. We also observed gradual balancing of the geographical distribution of *B*. *anthracis* population in northwestern China according to the canSNP analysis. In particular, the A.Br.Ames subgroup now seems to be scattered from Inner Mongolia to other provinces, in contrast to the data before 1990. This study also provides preliminary survey results on the population diversity of *B*. *anthracis* in China, which will help to promote the prevention and control of this important disease.

## Introduction

Anthrax, caused by the bacterium *Bacillus anthracis*, is primarily a disease in herbivores and sometimes sparks outbreaks in humans, with potentially serious consequences [1–4]. This disease is enzootic in most countries in Africa and Asia, as well as in some countries in Europe and America [4,5]. To date, anthrax remains an endemic disease, and human cases of this disease are reported every year in China, especially in northern and western provinces of China, such as Xinjiang, Shaanxi, Gansu, Inner Mongolia, Ningxia and Qinghai province [6]. Because of its wide distribution and its potential use for bioterrorism, anthrax is considered a global public health threat [7]. One of the most notorious bioterrorism events associated with *B*. *anthracis* was the “letter attacks” that occurred in the United States in 2001 [6]. In addition, the emergence of “injectional anthrax” among heroin users in Europe highlights the possibility of new routes for the spread of human anthrax [7,8].

*B*. *anthracis* is a genetically homogeneous pathogen. Currently, single-nucleotide polymorphism (SNP) analysis and multilocus variable-number tandem repeat analysis (MLVA) might be the most effective methods for genotyping *B*. *anthracis* [9–11]. An approach using 13 selected SNPs located at key phylogenetic junctions in the *B*. *anthracis* SNP tree, termed canonical single-nucleotide polymorphisms (canSNPs), are currently the most affordable first line genotyping assays [11]. The global genetic population structure of *B*.*anthracis* has also been subsequently defined by the canSNP and MLVA methods [11–13]. As source tracking methods in anthrax outbreaks, these methods have also been widely used to illustrate the phylogenetic relationships of *B*. *anthracis* at national or international levels [14–17]. In China, the strains mostly isolated from Xinjiang province in 1981/1982 were genotyped by these methods [18].

Since 2005, the Anthrax National Surveillance Project has been covering 12 provinces in mainland China. Additional human anthrax cases were reported and a series of *B*. *anthracis* strains were collected in the surveiled provinces. Concerns have been heightened by the persistence of human anthrax cases and outbreaks in certain areas in recent years, e.g., the outbreaks in Shaanxi in 2015, Xinjiang in 2016, Gansu in 2016 and Inner Mongolia in 2011 [19–21]. The northwestern provinces, namely, Xinjiang, Gansu, Qinghai, Ningxia, Shaanxi and Inner Mongolia, are the important disease-endemic areas, where almost half of human cases during 2005–2016 in China were found. In this study, the strains collected from 1990 to 2016 in northwestern China were investigated, and the genetic relationships among the strains were analyzed with the canSNP and MLVA methods.

## Methods

### Ethics statement

This study was reviewed and approved by the Ethics Committee [Institutional Review Board (IRB)] of the National Institute for Communicable Disease Control and Prevention, China CDC (license number: ICDC-2014013). All adult subjects provided informed consent, and a parent or guardian of any child under age18 participant provided informed consent on their behalf. The informed consent was given orally by all participants as this is standard practice in anthrax outbreak investigations, and oral consent is also a safe manner to minimize the risk of contamination since most patients have lesions on their arms or hands. The consent was recorded in daily progress notes by the attending physician at the local hospital. The IRB approved the use of oral consent, and the information provided to obtain consent included the aim of the study and the usage of the patients’ samples. No live animals were euthanized in this study, and the samples were collected from dead animals with permission from the owners of the animals.

### Epidemiologic data

The Anthrax National Surveillance Project is part of the Chinese Notifiable Disease Reporting System. Human anthrax surveillance data were from a real-time online nationwide reporting system, in which each human anthrax case was reported through a standardized form. We included in the analysis all human anthrax cases with illness onset during 2005 to 2016 in northwestern China. Descriptive statistics were used in the study.

### Bacterial strains

A total of 106 *B*. *anthracis* strains, including 13 DNA samples, collected from a variety of sources from six provinces in northwestern China, were included in this study. Eighty-eight of the strains (accounting for 83.02%) were collected from 1990 to 2016, and 18 strains (accounting for 16.98%) were collected before 1990. These strains were recovered from the following provinces, with the number of strains given in parentheses: Xinjiang (46), Gansu (9), Inner Mongolia (32), Qinghai (4) and Shaanxi (15); no strain was collected from Ningxia. The strains were obtained from the following sources: human (48), fur (10), sheep (9), cattle (6), soil (14), goat (1), dog (1), mules (5), yak(1) and unknown sources (11) (S1 Table). All of the strains were initially identified by *B*. *anthracis* species-specific gene targeting of the protective antigen gene (*pagA*, GenBank accession no. AE017336), capsule synthesis gene (*capC*, GenBank accession no. AE017335) and chromosomal *rpoB* gene (GenBank accession no. AE017334). To further understand the genetic characteristics of the pathogen, the variable number tandem repeat (VNTR) profiles and canSNP data associated with 133 *B*. *anthracis* strains collected in China in previous research were also merged into the analysis [18].

### DNA preparation

*B*. *anthracis* strains were streaked onto LB agar plates and incubated at 37°C for 16–18 h. Single colonies were then suspended in 0.5 ml of TE buffer (10.0mM Tris-HCl [pH 8.0], 1.0 mM EDTA) and incubated at 100°C for 20 min. Then, cellular debris were removed by centrifugation at 15,000×g for 10 min. The supernatant was collected and filtered using 0.22 μm filters. The filtered supernatant was diluted (1:10) with sterile nuclease-free H_2_O and used as the DNA template for PCR amplification. Bacterial culture growth and preparation of DNA samples were performed in a biosafety level 3 (BSL-3) laboratory.

### CanSNP genotyping

CanSNP analysis with the 13 markers was performed as described by Van Ert et al [11]. All amplifications were performed on a Roche LC480 instrument (Roche Diagnostics, Penzberg, Germany), and the cycling parameters were as follows: 95°C for 30 s; 45 cycles of 95°C for 5 s and 60°C for 20 s; and a final step at 40°C for 30 s. Each reaction included 10 μl of 2× reaction mix, each of a pair of primers at 0.2 μM, each of a pair of probes at 0.2 μM, and ddH_2_O for a final volume of 20 μl. The nomenclature and terminology of the sublineages in the canSNP analysis were consistent with the original literature. The A.Br.008/009 subgroup was subtyped by A.Br.011, which was performed as described by Marston et al [22].

### MLVA genotyping

Fifteen markers were used in this study for MLVA. These markers included eight markers initially described by Keim et al [6] and another seven markers described by Van Ert et al in 2007 [5]. The latter combined the 15 markers and devised the MLVA15 method. The MLVA15 was performed as described previously by capillary electrophoresis with the following modifications. The forward primers were labeled with different fluorescent dyes (vrrA, vrrB1, vrrB2, vrrC1, pXO1-aat, VNTR16, VNTR23, VNTR32 and VNTR35 labeled with Fam and the others labeled with Hex). PCR amplifications were performed on a SensoQuest Labcycler (SensoQuest, Germany) with an initial denaturation step at 95°C for 5 min, which was followed by 34 cycles of denaturation at 95°C for 30 s, annealing at 60°C for 30 s (52°C for vrrB1, vrrB2, VNTR12, VNTR16 and VNTR17) and extension at 72°C for 1 min. The reactions were terminated by a final incubation at 72°C for 5 min. The amplicons were diluted in water (1:100; for DNA templates from blister fluid samples from patients, the amplicons were not diluted), and after denaturation by heating, the amplicons were separated by capillary electrophoresis on an ABI 3730xl DNA analyzer with a GeneScan 1200 LIZ size standard (Applied Biosystems). The lengths of the amplicons were determined according to size using GeneMapper software V.4.0 (Applied Biosystems). Selected PCR products were sequenced to verify the tandem repeat sequences. The electrophoretic band sizes obtained in this study were corrected according to the sequencing results of the PCR products.

### Cluster analysis of the data

Data were imported into the BioNumerics software package (version 5.10, Applied Maths) as character data sets. Data from the canSNP analysis and MLVA were processed by cluster analysis using the categorical coefficient and the unweighted pair-group method with arithmetic means. Cluster analysis of the categorical data was presented using dendrograms.

## Results and discussion

### Anthrax in northwestern China

**Fig 1:**
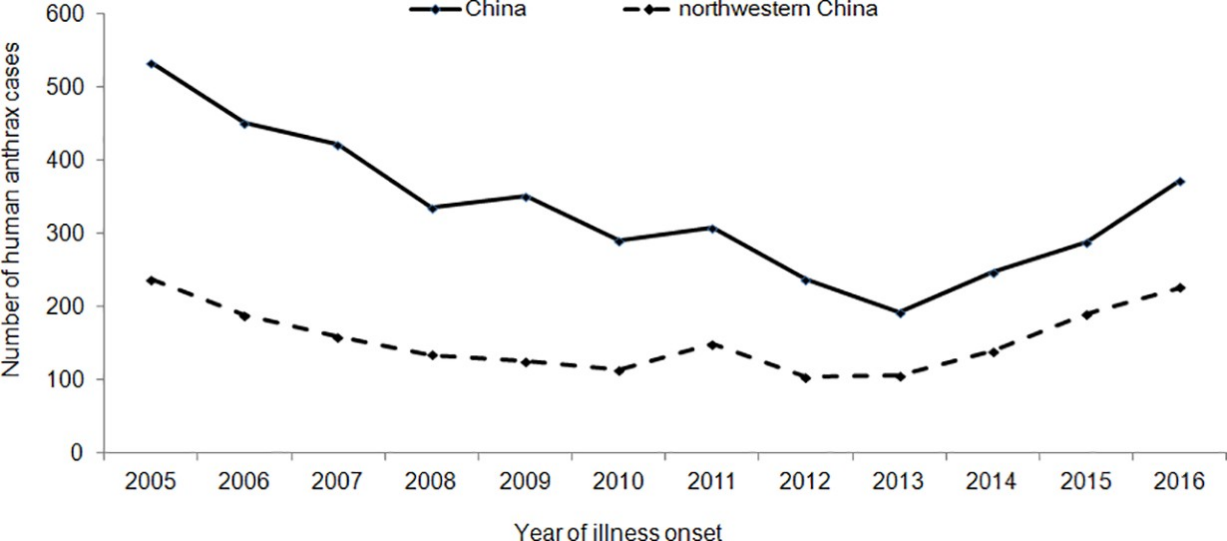
Annual distribution of human anthrax cases in northwestern China from 2005 to 2016.

According to the national epidemiological surveillance data, a total of 4030 human anthrax cases were reported in mainland China from 2005 to 2016. Approximately 47% of the cases were located in northwestern provinces (Table 1).The epidemic curve showed an overall decreasing trend from 2005 to 2013, increasing again after 2013, especially in the Gansu, Qinghai, and Inner Mongolia provinces. As a result, the cases of anthrax in the six provinces of northwest China accounted for 60.86% of the total in 2016 (Fig 1).

**Table 1:** Human anthrax cases in northwestern China, 2005–2016.

Year	Nationwide	Northwestern China	Shaanxi	Gansu	Qinghai	Ningxia	Xinjiang	Inner Mongolia
2005	533	238	8	64	25	20	76	45
2006	451	188	9	50	19	14	86	10
2007	421	159	3	43	43	2	52	16
2008	336	135	3	45	26	5	40	16
2009	351	127	2	46	17	6	42	14
2010	290	115	0	19	16	0	55	25
2011	309	149	1	39	23	0	45	41
2012	237	104	4	23	21	3	29	24
2013	193	107	3	41	36	0	22	5
2014	248	140	0	55	38	3	34	10
2015	288	191	24	70	59	3	13	22
2016	373	227	7	82	69	6	24	39

### Anthrax outbreaks in northwestern China in the last few years

In recent decade, human and livestock anthrax outbreaks have been reported in many provinces across China, such as Liaoning, Jiangsu, Yunnan and Xinjiang [23–26]. Since 2011, there have been four large-scale outbreaks in northwestern China, in 2011, 2015 and 2016 (Table 2). We conducted a comprehensive, in-depth retrospective epidemiological and molecular study for genetic source tracking of the *B*. *anthracis* species. From the four events, we observed that the cases all occurred in summer, June or August, and the infections originated from the slaughtering, skinning or butchering of sick animals, such as sheep, cattle, and mules. Previous studies concluded that anthrax in China was characterized by significant seasonality and spatial clustering [27], and we observed the same seasonality and animal exposure history (Table 2).

**Table 2:** Characteristics of anthrax outbreaks in northwestern China from 2011 to 2016.

Provinces	Time	Cases	Contacted ill animals	Strains or DNA samples	Genetic tracing		
					canSNP		MLVA15
Inner Mongolia	Aug.2011	39	Cattle	9	Ames, 001/002	CHN7, CHN18, CHN3*	
Shaanxi	Aug.2015	20	Mule	14	001/002	38	
Gansu	Aug.2016	21	Sheep Cattle	4	001/002	28, 30	
Xinjiang	Jun.2016	10	Cattle	1	Ames	31	

*For the new genotypes, the nomenclatures labeled “CHN” were organized in this study.

Two of the four outbreaks need to be highly concerned. The two outbreaks occurred in Ganquan County (Shaanxi, 2015) and Min County (Gansu, 2016), which were not historically high-risk areas of anthrax occurrence; the last anthrax case reported in Ganquan County was in 1973 and that in Min County was in 1958. A sudden rise in temperature and heavy rain were recorded in both of the afflicted regions in the year of the outbreak. A retrospective epidemiological study conducted by Chen WJ et al [27] showed that temperature, relative humidity and rainfall were positively correlated over time with human anthrax in the most likely clustering areas. Increased rainfall and temperature in the summer could unearth the anthrax spores and facilitate the breeding of the bacteria. The outbreak-related strains and samples were classified as the A.Br.001/002 subgroup, which is the most prevalent group in China. In Shaanxi, the samples collected from patients and mules were assigned to the MLVA15-38 genotype. In Gansu, one strain was assigned to MLVA 15–28 and the other three were assigned to MLVA 15–30.

On the other hand, anthrax outbreaks frequently occur in livestock and humans in the Inner Mongolia and Xinjiang provinces. The surveillance data showed that the two outbreak areas (Xing’anmeng in Inner Mongolia and Tuokexun County in Xinjiang) were high-epidemic regions, with cases reported every year, such as 23 cases in 2001, 14 in 2004 and 12 in 2010 in Tuokexun County. The outbreak-related strains were classified as subgroups A.Br.Ames and A.Br.001/002. This is the first time that the A.Br.Ames subgroup was found in Xinjiang. Using the MLVA15 scheme, we identified three new genotypes, MLVA15-CHN3, CHN7, and CHN18, in Inner Mongolia and MLVA15-31 in Xinjiang.

### CanSNP analysis of strains from northwestern China

The 106 *B*. *anthracis* strains (including 13 DNA samples) from the investigated provinces were distributed into 5 of the 12 canSNP sublineages/subgroups described by Van Ert et al [5]. These canSNP subgroups were A.Br.001/002 (n = 50), A.Br.Ames (n = 18), A.Br.008/009 (n = 11), A.Br.Vollum (n = 4) and A.Br.Aust94 (n = 23). A previous study by Simonson [18] described the same characteristics for *B*. *anthracis* collected from eight provinces in China (a total of 191 strains were included), but in that study, a majority of the strains were collected from Xinjiang, and those strains were all obtained before 1990. As a result, Xinjiang harbored 4 different sublineages of *B*.*anthracis* and Inner Mongolia harbored 2 sublineages, while only one sublineage, A.Br.001/002, was found in the other six provinces. In later studies, the A.Br.008/009 has been divided into A.Br.008/011 and A.Br.011/009, so we typed the 11 strains in the group with A.Br.011, all of them showed same characteristic and didn’t provide more helpful information for the study. So we still used the original nomenclature, because the objective of the study was mainly to compare the results with the previous study by Simonson. From our study, the same 5 canSNP lineages were found and the Xinjiang province is also the most diverse. However, the geographical distribution of the *B*. *anthracis* population showed different canSNP characteristics from the strains isolated before 1990 in China. The A.Br.Ames subgroup was first found in Xinjiang, and it seems that the subgroup was scattered from Inner Mongolia to other provinces, such as Xinjiang and Gansu. The A.Br.Aust94 subgroup was observed in Qinghai (2 strains from 1996) and Inner Mongolia (1 strain from 1997). The A.Br.Vollum and A.Br.008/009 subgroups were also observed in Qinghai (1 strain from 1996) and Inner Mongolia (1 strain from 1974), respectively. The new pattern may be a result of the isolation year, strain selection, and infection outbreak.

**Fig 2:**
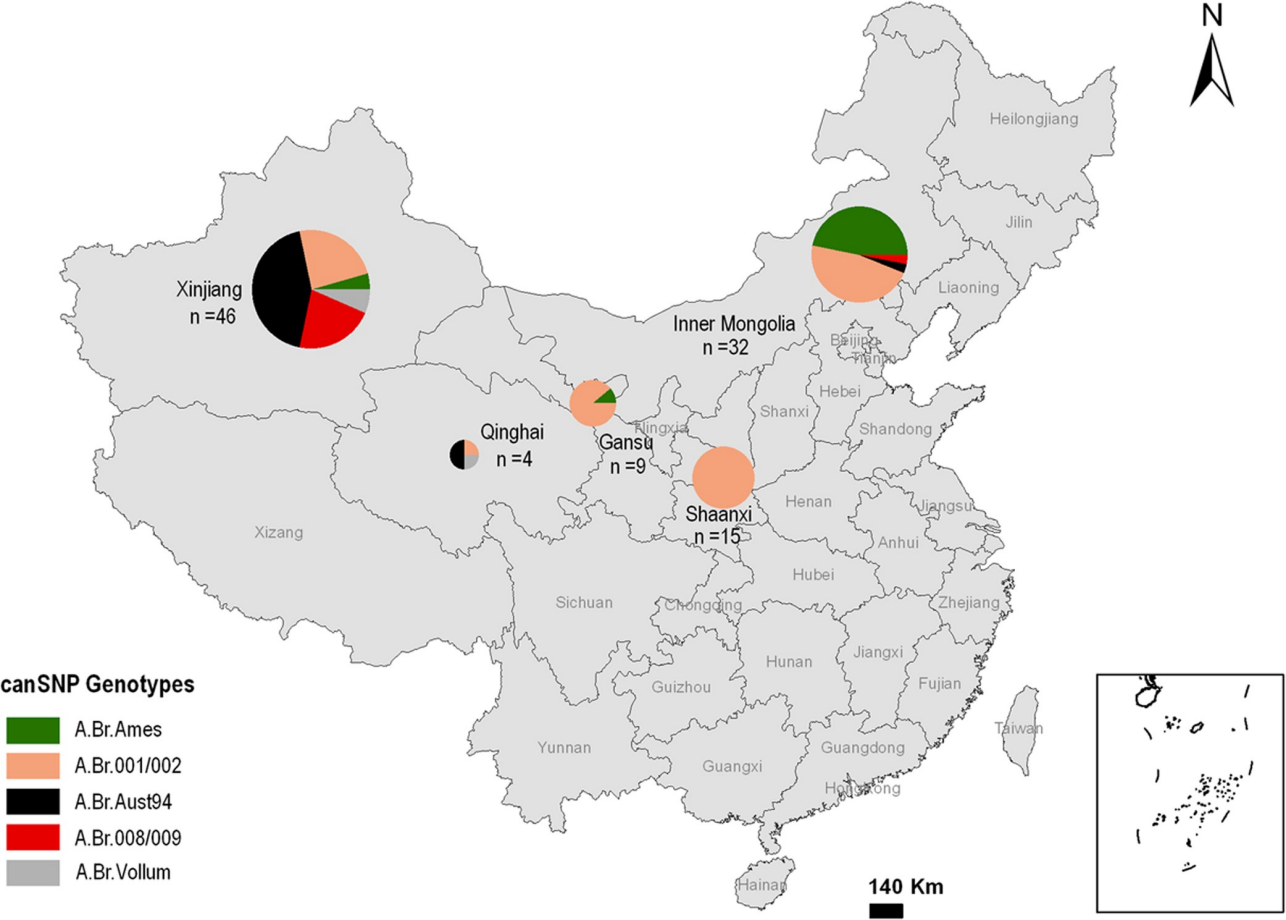
Geographical distribution of canSNP genotypes of *B*. *anthracis* strains from northern and western China. This distribution is based on 12 canSNP genotypes described by Van Ert et al [5] and the analysis of 106 strains from five provinces of China. The subgroups are indicated by different colors. The letter n represents the number of strains from the province. We used ArcGIS version 10.0 (ESRI, USA) and Photoshop CS 8.0.1 (Adobe Systems Incorporated, USA) to plot the maps.

Among the provinces, Inner Mongolia is dominated by the A.Br.Ames sublineage (15 of 18 strains). Gansu and Shaanxi are dominated by the A.Br.001/002 subgroup. The 4 strains collected in 1996 and 2005 from the Qinghai province were distributed into three subgroups, namely, A.Br.001/002, A.Br.Aust94 and A.Br.Vollum. Xinjiang was dominated by the A.Br.Aust94 sublineage and had 20 of 46 strains (43.48%). The A.Br.008/009 cluster also accounted for 10 strains in this province (21.74%). The above two subgroups(A.Br.Aust94 and A.Br.008/009) are spread across most of Europe and Asia. The A.Br.001/002 subgroup, which is mainly found in the remaining provinces of China, was also predominant in Xinjiang (23.91%, 11/46). The A.Br.Vollum sublineage accounts for only 3 strains in Xinjiang. The data from the Anthrax National Surveillance Project in mainland China showed that the Xinjiang province is a high-epidemic area, especially Kashi city, which is a major “oasis” city located at crossroads along the ancient Silk Road (dating back more than 2000 years) [28]. From 1990 to 1994, severe anthrax outbreaks occurred in this city. Two towns, Zepu and Atushi, recorded 24 villages with 202 human infections and 4 villages with 81 human infections, respectively [18]. A series of *B*. *anthracis* strains were collected, and most of the collection was included in this study. The genotypes A.Br.008/009, A.Br.Vollum and A.Br.Aust94, which are spread across most of Europe and Asia, were found only in Xinjiang according to the study described by Simonson[18]. Here, we observed that these genotypes were also found in Inner Mongolia and Qinghai, which suggested that the geographical distribution of the *B*. *anthracis* population seems to have gradually become more balanced from west to east, that is to say, different provinces tend to converge on the distribution of these subgroups (Fig 2).

### MLVA of strains from northwestern China

**Fig 3:**
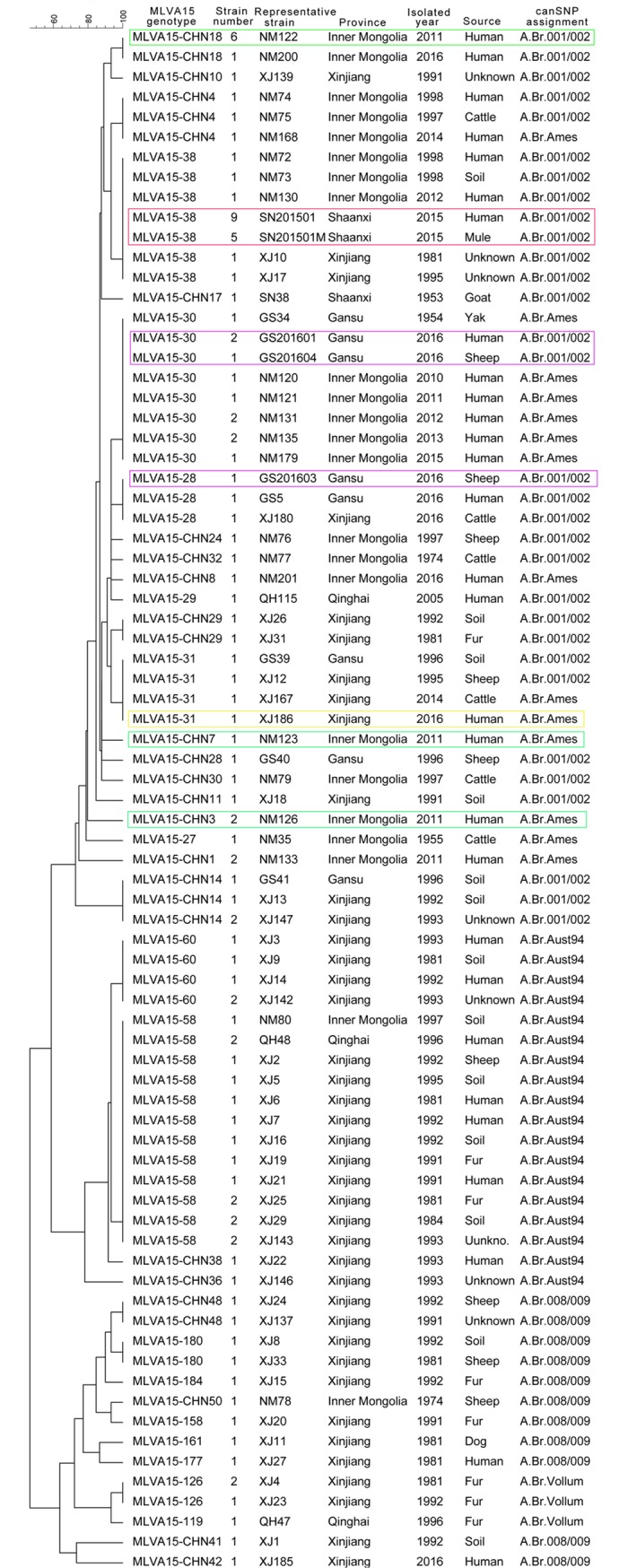
Dendrogram of canSNP typing and MLVA15 for the strains in northwestern China, during 1990–2016. The nomenclature of genotypes of MLVA15 according to Keim Genetics Lab ID Designation. For the new genotypes, the nomenclatures labeled “CHN” were organized in this study. MLVA genotyping showed much greater genetic diversity in A.Br.008/009 and A.Br.001/002 subgroups. For the outbreak-related strains, the red-framed strains are for Shaanxi, the green-framed are for Inner Mongolia, the blue-framed are for Gansu and the yellow-framed is for Xinjiang.

*B*. *anthracis* spores can persist for long periods of time in the environment, and this distinct life cycle allows *B*. *anthracis* to be characterized by relatively few genetic variations [11]. The canSNP analysis, with low resolution, is not adequate for the investigation of an infectious source. A scheme combining canSNP analysis and MLVA15 was used to analyze anthrax outbreaks in many countries, which facilitated the employment of a genetic population structure comparison [14,16,17]. In China, recently, similar methods were used to trace the source of outbreaks in Liaoning and Shaanxi provinces [19,29]. In this study, subtyping using the MLVA15 scheme indicates that all of the 106 strains were clustered into 36 MLVA genotypes. We found 21 new MLVA genotypes, which were named MLVA15-CHN (S1 Table and Fig 3).

The results showed the gradual diversification of MLVA subtyping patterns of *B*. *anthracis* with the development of the anthrax outbreaks. There were 8 MLVA genotypes in A.Br.008/009 for 11 strains, 4 in A.Br.Aust94 for 23 strains, 16 in A.Br.001/002 for 50 strains, 8 in A.Br.Ames for 18 strains and 2 in A.Br.Vollum for 4 strains. The genetic diversity of the A.Br.008/009 and A.Br.001/002 subgroups suggests repeated infections and outbreaks for the subgroups of *B*. *anthracis* and a significantly longer history for this particular clade in the studied region. It has been known that distant transportation of livestock can cause the spread of anthrax; for example, in 2012, a human anthrax outbreak occurred in Lianyungang, Jiangsu province, where no anthrax cases had been reported for many years, and epidemiological investigation found that sick cattle transported from Liaoning province were the source of the infection [29]. Similarly, the outbreak in Min County, Gansu province in 2016 was possibly due to a livestock trade fair held in this county in 2016, when a batch of yak were brought from Qinghai and Sichuan. The genotypes of outbreak-related strains were MLVA 15–30 and MLVA15-28, and the latter genotype was the same as that of a strain isolated from Xinjiang in 2016. The outbreak-related strains in Ganquan, Shaanxi province were assigned to MLVA15-38, which was the same as the strains from Xinjiang (2 strains in 1981 and 1995) and Inner Mongolia (2 strains in 1998 and one in 2012). Based on the results of the epidemiological investigation, we speculated that the outbreak-related strains may originate from Inner Mongolia. In addition, significant differences were not observed between the year of isolation from the 1980s and 1990s. This observation indicated that the strains attributed to the anthrax outbreaks that occurred in 1990s may be similar to the strains from the 1980s.

Even though the MLVA showed a relatively high resolution, this method did not always meet the requirements for detailed source tracking of outbreaks. SNR (single-nucleotide repeat) markers could provide additional genetic resolution among *B*.*anthracis* strains of the same MLVA genotype and has been used in molecular investigation of anthrax epidemics or outbreaks[30,31].

Although the combination of the canSNP and MLVA used in the study has been a useful tool for molecular epidemiology investigation and source tracking, it couldn’t provide enough information for phylogenetic analysis. Whole genome sequence analysis is increasingly becoming a standard method in terms of phylogenetic investigations for clonal bacterial species such as *Bacillus anthracis*. Owing to rapid progress in this field, the approach would be a more powerful tool in future.

## Supporting information

S1 Checklist STROBE checklist. (DOC)

S1 Table *B*. *anthracis* strains used in this study and the MLVA15 data. (XLSX)
